# Strange Days: Adult Physical Activity and Mental Health in the First Two Months of the COVID-19 Pandemic

**DOI:** 10.3389/fpubh.2021.567552

**Published:** 2021-04-15

**Authors:** Madelaine Gierc, Negin A. Riazi, Matthew James Fagan, Katie M. Di Sebastiano, Mahabhir Kandola, Carly S. Priebe, Katie A. Weatherson, Kelly B. Wunderlich, Guy Faulkner

**Affiliations:** Population Physical Activity Lab, School of Kinesiology, University of British Columbia, Vancouver, BC, Canada

**Keywords:** depression, anxiety, life satisfaction, public health, moderate-to-vigorous physical activity

## Abstract

**Background:** In addition to its physical health benefits, physical activity is increasingly recognized as a means to support mental health. Regular moderate-to-vigorous physical activity (MVPA) is associated with improved mental well-being, reduced likelihood of developing mental illness, and improved symptom management. Despite these benefits, most people fail to achieve minimum recommended levels of MVPA. Population levels of physical activity have further declined since the onset of the COVID-19 pandemic and implementation of public health measures (e.g., shelter-in-place protocols). The potential impact of this decline on mental heath outcomes warrants ongoing investigation.

**Purpose:** To investigate associations between changes in MVPA and mental health (depressive symptoms, anxiety symptoms, and life satisfaction) in adults impacted by the COVID-19 pandemic.

**Method:** Research followed a cross-sectional design. English-speaking adults were invited to complete an online questionnaire. MVPA was assessed retrospectively (before COVID-19) and currently (during COVID-19) with the International Physical Activity Questionnaire. Mental health was assessed with the Patient Health Questionnaire, 9-Item (PHQ-9), the Generalized Anxiety Disorder, 7-Item (GAD-7), and the Satisfaction with Life Scale (SWLS). Regression was used to assess relationships between MVPA and mental health. ANOVA with follow-up tests examined whether participants who differed in mental health status (e.g., no symptoms vs. severe symptoms) differed in their change in MVPA. *T*-tests were used to examine differences in mental health symptomatology between participants who were sufficiently (i.e., achieving MVPA guidelines of ≥ 150 min/week) vs. insufficiently active.

**Results:** Prior to COVID-19, 68.2% of participants were classified as being sufficiently active, vs. 60.6% during COVID-19. The majority of participants reported experiencing some level of depressive symptoms (62.0%) or anxiety symptoms (53.7%). After controlling for covariates, changes in MVPA accounted for significant variability in the PHQ-9 (7.7%), GAD-7 (2.5%), and SWLS (1.5 %). Participants with clinically significant mental health symptomatology reported greater declines in MVPA than those who reported no symptoms. Conversely, participants who were sufficiently active during COVID-19 reported significantly lower depression and anxiety, and higher life satisfaction.

**Conclusion:** Participants who experienced the greatest declines in MVPA reported relatively greater psychological distress and lower life satisfaction. While preliminary, these findings suggest the importance of maintaining and promoting physical activity during a period of pandemic.

## Introduction

Regular participation in physical activity has long been recognized for its physical health benefits. For instance, moderate-to-vigorous physical activity (MVPA; e.g., brisk walking, lap swimming) is known to both prevent and manage chronic conditions like cardiovascular disease, type-2 diabetes, and cancer (1); whereas balance/flexibility activities (e.g., yoga, tai chi) are associated with improved mobility and functional abilities (2–4). Similarly, physical activity is increasingly embraced as a means through which to support mental health. Not only does physical activity promote well-being (5), but it is associated with reduced risk of developing mental illness (6) and improved symptom management in those with a pre-existing condition (7). The benefits of physical activity have been observed across diverse psychiatric and neurological conditions, such as anxiety (8), post-traumatic stress disorder (9), attention-deficit hyperactivity disorder (10), and dementia (11, 12). Recently, exercise has been recommended as a first-line treatment for mild-to-moderate depression and as an adjunctive treatment of moderate-to-severe depression (13).

Various national (14, 15) and international (16) physical activity guidelines recommend that adults engage in at least 150 min of MVPA per week to achieve health benefits. Muscle strengthening and balance/flexibility activities are also recommended. Surveillance studies consistently illustrate that populations fail to achieve this minimum level of activity. In the United States, ~23% of adults meet both aerobic and muscle-strengthening physical activity guidelines (17). Similarly, in a device-based study of Canadians, only 15% of participants achieved national physical activity guidelines, whereas only 5% achieved them on a regular basis (18). Such low levels of PA have notable implications for the health and wellness of nations.

### Physical Activity in the Time of COVID-19

The novel coronavirus disease, COVID-19 (“COVID”), was first identified in the Wuhan region of China in December 2019 (19, 20). It was subsequently declared a global pandemic on March 13, 2020. In an effort to control the spread of the virus, many nations have implemented widespread and significant public health measures such as closing non-essential businesses, closing international borders, banning large gatherings of people, mandatory self-isolation, and requiring individuals to maintain a minimum physical distance with others.

An inadvertent consequence of COVID public health protocols has been a decline in physical activity. A systematic review of 66 studies examining physical activity and sedentary behavior during COVID demonstrated consistent declines in physical activity during the initial COVID-19 lockdown regardless of subpopulation or methodology used (21). Such observations are not unexpected given widespread closure of recreation facilities and public parks, a shift to working from home, and shelter-in-place protocols. In Canada, declines of ~13, 15, and 13% were observed in device recorded MVPA, light physical activity, and step counts, respectively (22). Though public health measures are necessary for reducing disease transmission, there is concern that the resulting reductions in physical activity may have implications for physical and mental health (23, 24). In response, individuals have been encouraged to remain active during the COVID pandemic (25–27), such as through outdoor exercise or home-/apartment-friendly activities (28, 29).

### Physical Activity, Mental Health, and COVID

The existent evidence for the mental health benefits of physical activity stems from studies that have been conducted under “regular conditions.” The unique characteristics of the COVID pandemic – including the rapid speed at which changes are occurring and the disruption in regular daily routines – are unprecedented in their scope and impact. Literature from early in the COVID pandemic indicates that symptoms of anxiety and depression (16–28%) and stress (8%) are common psychological reactions to COVID (30). The extent to which physical activity may buffer against the psychosocial impact of COVID remains less studied. In Canada, individuals who were inactive during the COVID pandemic reported lower mental well-being and higher anxiety compared to active individuals (31) and women reported significantly higher generalized anxiety than men (32). The purpose of the current study was to extend this work and examine associations between MVPA and other mental health outcomes in the early phases of the COVID pandemic. It was hypothesized that a negative relationship would be observed between changes in MVPA and psychological distress (anxiety and depressive symptomatology); and that a positive relationship would be observed between changes in MVPA and life satisfaction.

## Materials and Methods

### Participants and Design

The current study followed a cross-sectional, observational design. All study protocols received approval from The University of British Columbia Research Ethics Board (#H20-00899). Individuals were eligible to participate if they were age 18 years or older and able to communicate in English. Given the classification of COVID as a pandemic at the time of study launch, no restrictions were placed on individuals' country of residence. With an alpha level of 0.05, power of 80%, and anticipating a small effect size (33), a total of 395 participants were required for regression analyses and 302 participants for ANOVA (34).

### Measures

#### Sociodemographic Variables

Basic sociodemographic information, such as educational attainment and employment status, were collected for descriptive purposes. Additionally, age, gender, and body mass index (BMI; from self-reported height and weight) were collected to serve as covariates in regression analyses (35–38).

#### Physical Activity

Self-reported MVPA was assessed with a modified International Physical Activity Questionnaire, Short Form (IPAQ) (39). The IPAQ is a validated measure of physical activity that is commonly used in epidemiological studies. The questionnaire invites participants to report the number of days per week and the amount of time per day (in hours and minutes) spent in vigorous physical activity, moderate physical activity, and light physical activity. Weekly minutes of vigorous- and moderate- intensity physical activity were calculated by multiplying by daily minutes by number of days. Subsequently, weekly minutes of MVPA were calculated by adding minutes of moderate-intensity and vigorous-intensity activity, with maximum scores truncated to 1260 min as guided by IPAQ scoring protocols (https://sites.google.com/site/theipaq/scoring-protocol). In the current study, participants reported physical activity twice: before COVID (“pre-COVID”) and over the past 7 days (“during-COVID”). For the pre-COVID measure, participants were asked to report their level of activity on a typical week *before* COVID-related restrictions were implemented. Participants were reminded that COVID was declared a pandemic on March 13, 2020; and that their reference period would likely be late February or early March.

#### Depressive Symptomatology

Depressive symptomatology over the last 2 weeks was assessed with the Patient Health Questionnaire, 9-Item (PHQ-9) (40). The PHQ-9 has nine items, which correspond with the criteria for major depressive disorder as outlined in the Diagnostic and Statistical Manual of Mental Disorders (DSM) (41). Responses are made on a 0 to 3 scale (maximum score of 27), with greater values indicating greater symptom severity. Scores of 5, 10, 15, and 20 are indicative of mild, moderate, moderate-severe, and severe major depressive symptoms, respectively. Using a threshold of ≥10, the scale has 0.88 sensitivity and 0.88 specificity for major depressive disorder (40).

#### Anxiety Symptomatology

The occurrence of general anxiety symptoms over the last 2 weeks was assessed with the Generalized Anxiety Disorder, 7-Item (GAD-7) (42). The GAD-7 has 7 items which correspond with the symptom criteria for generalized anxiety disorder as outlined in the DSM (41). Like the PHQ-9, responses are made on a 0 to 3 scale (maximum score of 21), with greater values indicating greater symptom severity. Scores of 5, 10, and 15 are indicative of mild, moderate, and severe anxiety symptoms, respectively. Using a threshold of ≥10, the scale has 0.89 sensitivity and 0.82 specificity for generalized anxiety disorder (42).

#### Life Satisfaction

Participants' overall sense of well-being was assessed with the Satisfaction with Life Scale (SWLS) (43). The SWLS was developed as a measure of the cognitive component of subjective well-being: that is, an individual's judgement regarding whether their life is good or poor. Participants are presented with 5 items, such as “The conditions of my life are excellent.” Items are rated on a five-point Likert scale, ranging from 1 (Strong Disagree) to 5 (Strongly Agree), with higher scores indicative of greater perceived quality of life. Though the scale is recommended for use as a continuous variable, it is also possible to score categorically (44) with classifications ranging from extremely satisfied (scores of 31–35) to extremely dissatisfied (scores of 5–9).

### Procedure

Participants were recruited online through social media advertisements (Twitter, Facebook, Instagram, LinkedIn) and alumni newsletters. Recruitment occurred between March 24 and May 8, 2020. Interested volunteers were directed via hyperlink to an online survey. After completing eligibility items and providing informed electronic consent, participants were presented with three clusters of questionnaire items: sociodemographic variables, the IPAQ, and the three mental health scales. At the end of the questionnaire, participants were debriefed, thanked for their participation, and were provided the opportunity to enter their name into a draw.

### Data Analysis

Data screening and management procedures were conducted in accordance with the recommendations of Tabachnick and Fidell (45). All analysis was conducted using SPSS Statistics, v.25 (IBM, US). Two groups of analyses were conducted. The first utilized hierarchical regression to examine associations between MVPA and mental health. Age, gender, and BMI were entered as covariates (35–38). Change in self-reported MVPA, calculated as the difference between during-COVID MVPA and pre-COVID MVPA, served as the predictor variable. Thus, negative values indicate a decrease in MVPA whereas positive values indicate an increase in MVPA. The outcome variables were PHQ-9, GAD-7, and SWLS continuous scores.

The second group of analyses examined between-group differences. With regards to mental health, participants were categorized according to standard interpretive thresholds for the PHQ-9 (40), GAD-7 (42), and SWLS (44). ANOVA was used to examine between-group differences in change in MVPA. With regards to MVPA, participants were classified as being sufficiently active if they self-reported ≥150 min MVPA per week, and insufficiently active if they reported <150 min MVPA (14–16). ANOVA was used to examine between-group differences in mental health outcomes (PHQ-9, GAD-7, SWLS) between these groups. Analyses were conducted twice, to examine pre-COVID and during-COVID MVPA status. Bonferroni correction was applied to control for Type I error. In all analyses, equal variance was not assumed.

## Results

### Participants

The online survey was accessed 1,005 times, of which 9 individuals were ineligible due to being age 17 or younger; 9 individuals were ineligible due to not being able to communicate in English; and five individuals declined consent to participate. A further 340 did not proceed beyond the eligibility screening questionnaire, primarily due to “bot” traffic. A total of 665 individuals accessed the intake survey, of which 248 provided incomplete data. There were no differences in survey completion based on age, gender, or BMI (*t* = 0.367, *p* = 0.713; *t* = 0.344, *p* = 0.731; and *t* = 1.814, *p* = 0.07, respectively).

The final sample had an average age of 32.2 (*SD* = 13.6) years. Most participants self-identified as being a woman (86.8%) and well-educated (62.7% with an undergraduate degree or greater), and were employed either full- or part-time (42.3%). The majority were classified in the normal range of BMI (62.7%). One-third (32.3%) of the sample identified they were of North American descent, such as Quebecois or American. Full sample details can be found in Table 1.

**Table 1:** Demographic and descriptive characteristics of participants.

		***N* (%)**	**Mean (sd)**
Age			32.2 (13.6)
BMI category			
	Underweight	1 (2.4)	
	Normal weight	322 (62.7)	
	Overweight	116 (22.6)	
	Obese	74 (14.4)	
Country			
	Australia	1 (0.2)	
	Canada	398 (79.8)	
	Hong Kong	1 (0.2)	
	Ireland	1 (0.2)	
	Philippines	17 (3.4)	
	UK/N.IRL	16 (3.2)	
	USA	65 (13.1)	
Gender			
	Woman	452 (86.8)	
	Man	66 (12.6)	
	Non-binary	2 (0.8)	
	Prefer not to answer	2 (0.4)	
Ethnicity			
	Indigenous	16 (3.0)	
	African	1 (0.2)	
	Central Asian	3 (0.6)	
	East Asian	129 (24.1)	
	Hispanic	21 (3.9)	
	Mediterranean	2 (0.4)	
	Middle Eastern	24 (4.5)	
	Pacific Islander	15 (2.8)	
	South American	1 (0.2)	
	South Asian	19 (3.6)	
	Northern European	59 (11.0)	
	Eastern European	6 (1.1)	
	Northern American	173 (32.3)	
	Western European	61 (11.4)	
	Other	1 (0.2)	
	Prefer not to answer	1 (0.2)	
Highest level of education			
	Less than high school	1 (0.2)	
	High School	98 (18.8)	
	Diploma or certificate	61 (11.7)	
	Undergraduate	196 (37.6)	
	Graduate	137 (26.3)	
	Professional degree	27 (5.2)	
	Prefer not to answer	1 (0.2)	
Employment Status			
	Student	168 (25.8)	
	Full time	199 (30.5)	
	Part time	77 (11.8)	
	Self-employed part-time	24 (3.7)	
	Retired	8 (1.2)	
	Homemaker	37 (5.7)	
	On disability	37 (5.7)	
	Unemployed looking for work	5 (0.8)	
	Unemployed not looking for work	2 (0.4)	
	Unemployed laid off	71 (10.9)	
	Other	22 (3.4)	
	Prefer not to answer	1 (0.2)	
Living location			
	City/urban	354 (68.1)	
	Suburbs	73 (14.0)	
	A town or village	39 (7.5)	
	Country/rural	54 (10.4)	
Type of housing			
	Detached	272 (52.1)	
	Semi-detached	63 (12.1)	
	Apartment/condo	92 (17.6)	
	Shared housing	76 (14.6)	
	A dormitory	18 (3.5)	
	Prefer not to answer	1 (0.2)	

*BMI, body mass index; SD, standard deviation*.

### Physical Activity

Participants self-reported a mean of 406 (*SD* = 380) minutes of MVPA per week prior to COVID, compared to 361 (*SD* = 388) minutes of MVPA per week after COVID. The mean reported change in MVPA was −45 (*SD* = 389) minutes. Approximately half of the sample (45.4%) reported a decrease in MVPA, while a quarter (22.7%) reported no change in MVPA, and the remainder (31.8%) reported an increase in MVPA.

Prior to COVID, 68.2% of participants self-reported being sufficiently active for health benefits (i.e., >150 min 193 MVPA per week). This decreased to 60.6% of participants during the initial period of the COVID pandemic.

### Mental Health

Participants reported a mean PHQ-9 score of 7.4 (*SD* = 6.0), indicative of mild depressive symptomatology. While a large minority (38.0%) reported experiencing no significant depressive symptoms, 33.9% reported mild symptoms, 14.4% moderate, 8.4% moderate-severe, and 5.3% severe. Similarly, participants reported a mean GAD-7 score of 6.5 (*SD* = 5.6). A large minority (46.3%) reported experiencing no significant anxiety symptoms, whereas 27.2% reported mild symptoms, 14.9% moderate, and 11.6% severe. With regards to well-being, participants reported a mean SWLS score of 24.1 (*SD* = 6.8). The majority (76.4%) of participants reported scores at or above the scale midpoint.

### Regression Models

All regression models controlled for age, gender, and BMI. Three separate models were examined: the relationship between (1) MVPA and PHQ-9, (2) MVPA and GAD-7, and (3) MVPA and SWLS.

Overall, the models provide evidence for associations between MVPA and mental health during COVID. For depression and anxiety, a self-reported decrease in MVPA was associated with higher scores on the PHQ-9 and GAD-7 (Pearson correlation = −0.284, *p* < 0.001; and −0.161, *p* = 0.001 respectively). Increases in MVPA were associated with higher levels of life satisfaction (Pearson correlation = 0.126, *p* = 0.007). Please see Table 2 for correlation tables for all variables included in models.

**Table 2:** Correlations between mental health, gender, age, BMI, and change in MVPA.

	**PHQ-9**	**Gender**	**Age**	**BMI**	**MVPA (change)**
PHQ-9	1.00				
Gender	0.022	1.00			
Age	−0.348^*^	0.111^*^	1.00		
BMI	0.87^*^	0.117^*^	0.159^*^	1.00	
MVPA (change)	−0.284^*^	−0.067	0.008	−0.007	1.00
	**GAD-7**	**Gender**	**Age**	**BMI**	**MVPA (change)**
GAD-7	1.00				
Gender	−0.01	1.00			
Age	−0.267^*^	0.111^*^	1.00		
BMI	0.076	0.117^*^	0.159^*^	1.00	
MVPA (change)	−0.161^*^	−0.067	0.008	−0.007	1.00
	**SWLS**	**Gender**	**Age**	**BMI**	**MVPA (change)**
SWLS	1.00				
Gender	−0.033	1.00			
Age	0.288^*^	0.111^*^	1.00		
BMI	0.042	0.177^*^	0.159^*^	1.00	
MVPA (change)	0.126^*^	−0.067	0.008	−0.007	1.00

* *Significant with Bonferroni correction*.

For mental health, significant variability was explained by change in MVPA from pre-COVID to during-COVID. After controlling for age, gender, and BMI, changes in MVPA accounted for 7.7 % variability in PHQ-9 scores (*F* = 26.182, *R* = 0.470, *adjusted R2* = 0.212, *R2 change* = 0.077, *p* < 0.001), 2.5 % variability in GAD-7 scores (*F* = 11.478, *R* = 0.333, *adjusted R2* = 0.101, *R2 change* = 0.025, *p* = 0.001), and 1.5 % variability in SWLS scores (*F* = 6.960, *R* = 0.265, *adjusted R2* = 0.060, *R2 change* = 0.015, *p* = 0.017). For the full model statistics, please see Table 3.

**Table 3:** Results of hierarchical regression analyses, examining associations between change in MVPA and mental health outcomes.

**Model**	**R**	**R Square**	**Adjusted R Square**	**Std. Error of the estimate**	**Change Statistics**				
					**R Square Change**	**F Change**	**Df1**	**Df2**	**Sig. F change**
**PHQ-9**									
1	0.379^a^	0.144	0.137	5.47	0.144	20.784	3	371	<0.001
2	0.470^b^	0.212	0.212	5.23	0.077	36.423	1	370	<0.001
**GAD-7**									
1	0.293^a^	0.086	0.079	5.378	0.086	11.595	3	370	<0.001
2	0.333^b^	0.111	0.101	5.312	0.025	10.257	1	369	0.001
**SWLS**									
1	0.235^a^	0.055	0.048	6.483	0.055	7.256	3	371	<0.001
2	0.265^b^	0.070	0.060	6.442	0.015	5.791	1	370	0.017

a Predictors (Age, Gender, BMI).

b *Predictors (Age, Gender, BMI, MVPA (change)*.

### Mental Health Status and Change in MVPA

**Figure 1:**
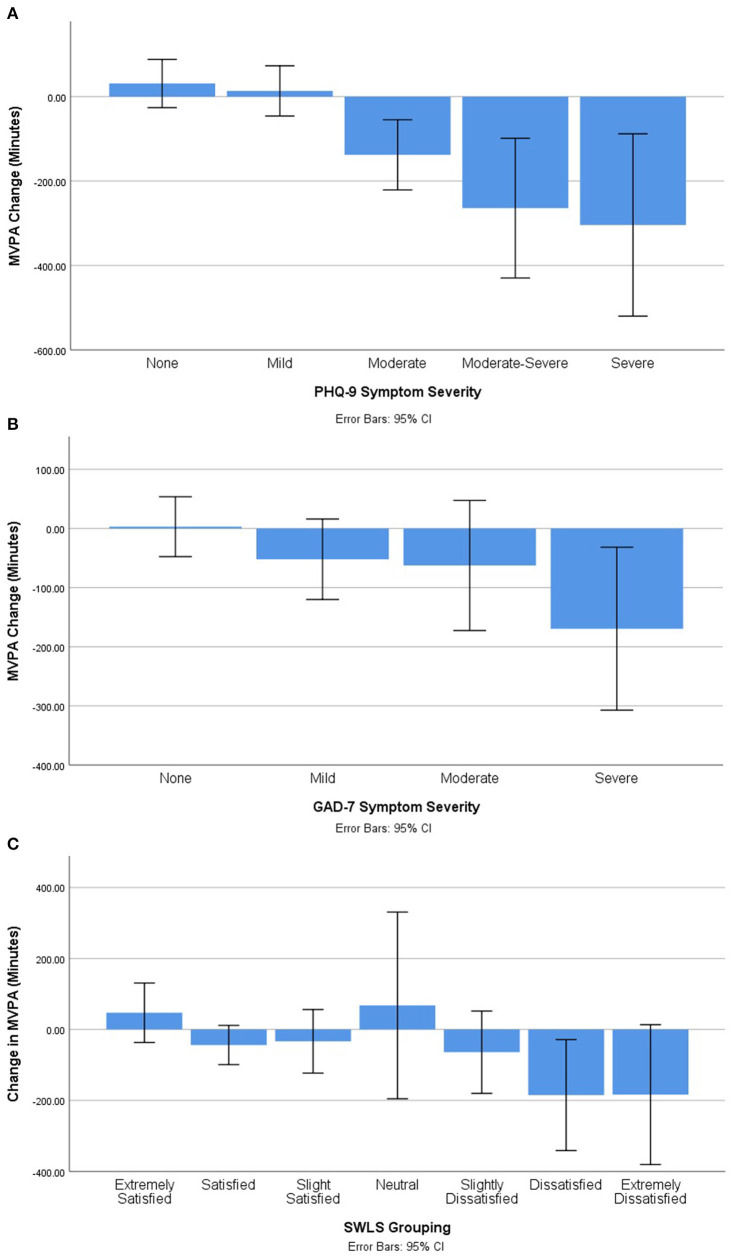
Change in self-reported MVPA by mental health status. **(A)** Self-reported change in MVPA by depressive symptom severity. **(B)** Self-reported change in MVPA by anxiety symptom severity. **(C)** Self-reported change in MVPA by life satisfaction grouping.

ANOVAs were conducted to examine whether degree of mental health symptomatology (e.g., none, mild, moderate, or severe) was associated with the extent to which MVPA changed from pre-COVID to during-COVID. In all analyses, significant differences were found. Bonferroni corrected *post-hoc* testing uncovered differences within the classifications of depressive symptoms, anxiety symptoms, and life satisfaction. Graphs displaying between-group differences can be found in Figure 1.

#### PHQ-9

For the PHQ-9, significant between-group differences were found (*F* = 62.206, *p* < 0.001). Follow-up analysis indicated significant differences between participants with no symptoms relative to those with mild (*MD* = −155.730, *SE* = 45.256, *p* = 0.006), moderate (*MD* = 286.193, *SE* = 41.403, *p* < 0.001), moderate-severe (*MD* = −285.345, *SE* = 69.902, *p* = 0.001), and severe symptomatology (MD = 718.765, SE = 61.576, *p* < 0.001).

Additionally, significant between-group differences were found between PHQ-9 severity of mild in comparison to moderate (*MD* = 441.923, *SE* = 50.438, *p* < 0.001) and severe (*MD* = 874.495, *SE* = 67.689, *p* < 0.001). There were also significant between-group differences found between PHQ-9 severity of moderate in comparison to moderate-severe (*MD* = −571.538, *SE* = 73.094, *p* < 0.001) and severe (*MD* = 432.571, *SE* = 65.176, *p* < 0.001). Finally, there was also a significant between-group difference found between PHQ-9 severity of moderate-severe in comparison to severe (*MD* = 1004.110, *SE* = 86.140, *p* < 0.001).

#### GAD-7

For the GAD-7, significant between-group differences were found (*F* = 4.026, *p* = 0.008). Follow-up analyses indicated significant differences between participants with no anxiety symptoms relative to those with mild (*MD* = 137.031, *SE* = 49.703, *p* < 0.037) and moderate symptoms (*MD* = 186.781, *SE* = 68.671, *p* = 0.041).

#### SWLS

In terms of the SWLS, significant between-group differences were found (*F* = 13.840, *p* < 0.001). Follow-up analyses identified significant difference between those who were extremely satisfied in comparison to satisfied (*MD* = 327.472, *SE* = 84.411, *p* = 0.004), neutral (*MD* = 632.609, *SE* = 103.992, *p* < 0.001), slightly dissatisfied (*MD* = 458.609, *SE* = 95.646, *p* < 0.001), dissatisfied (*MD* = 446.894, *SE* = 99.916, *p* < 0.001) and extremely dissatisfied (*MD* = 842.609, *SE* = 123.199, *p* < 0.001).

Additionally, significant between-group differences were found between the SWLS rating of satisfied in comparison to neutral (*MD* = 305.137, *SE* = 73.348, *p* < 0.001) and extremely dissatisfied (*MD* = 515.137, *SE* = 98.709, *p* < 0.001). Significant between-group differences were found between the SWLS rating of slightly satisfied and neutral (*MD* = 408.500, *SE* = 76.468, *p* < 0.001), slightly dissatisfied (*MD* = 234.500, *SE* = 64.662, *p* = 0.007), dissatisfied (*MD* = 222.786, *SE* = 70.825, *p* = 0.037) and extremely dissatisfied (*MD* = 618.500, *SE* = 101.050, *p* < 0.001). Significant between-group differences were found between the SWLS rating of slightly dissatisfied and extremely dissatisfied (*MD* = 384.000, *SE* = 106.887, *p* = 0.008). Finally, a significant between-group difference was found between SWLS rating of dissatisfied and extremely dissatisfied (*MD* = 395.714, *SE* = 110.724, *p* = 0.008).

### MVPA Guidelines and Mental Health

**Figure 2:**
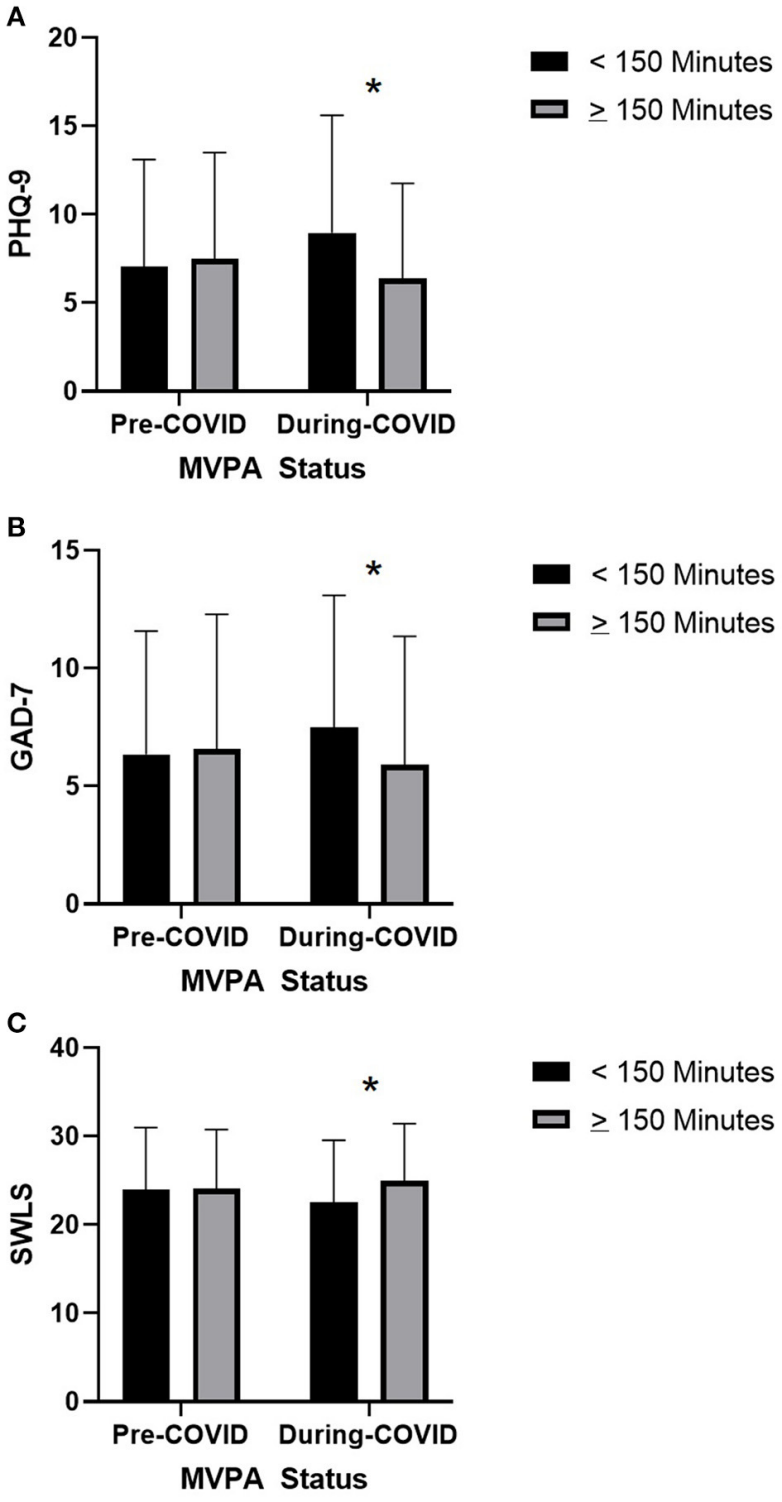
Associations between pre- and during-COVID MVPA status and mental health symptoms. **(A)** Associations between pre- and during-COVID MVPA status and during-COVID depressive symptoms. **(B)** Associations between pre- and during-COVID MVPA status and during-COVID anxiety symptoms. **(C)** Associations between pre- and during-COVID MVPA status and during-COVID life satisfaction. *Significant with Bonferroni correction.

*T*-tests were completed to determine if differences in total score of mental health measures (PHQ-9, GAD-7, SWLS) existed between individuals who were sufficiently active for health benefits vs. those who were insufficiently active. Analyses were conducted twice, on pre-COVID and during-COVID levels of activity. Graphs displaying between-group differences can be found in Figure 2.

#### PHQ-9

Participants who were sufficiently vs. insufficiently active pre-COVID did not significantly differ in their during-COVID PHQ-9 scores (*t* = 1.234, *df* = 288.272, *p* = 0.218, *MD* = 0.800). However, significant differences were observed between individuals who were sufficiently vs. insufficiently active during COVID (*t* = 13.400, *df* = 237.514, *p* < 0.001, *MD* = 8.074), with sufficiently active individuals reporting lower PHQ-9 scores.

#### GAD-7

Participants who were sufficiently vs. insufficiently active pre-COVID did not differ in their during-COVID GAD-7 scores (*t* = 3.427, *df* = 165.512, *p* = 0.001, *MD* = 2.372). Significant differences were observed between individuals who were sufficiently vs. insufficiently active during COVID (*t* = 10.907, *df* = 238.454, *p* < 0.001, *MD* = 5.706), with sufficiently active individuals reporting lower GAD-7 scores.

#### SWLS

Participants who were sufficiently vs. insufficiently active pre-COVID did not differ in their during-COVID SWLS scores (*t* = −1.335, *df* = 180.246, *p* = 0.183, *MD* = −1.026). Significant differences were observed between individuals who were sufficiently vs. insufficiently active during COVID (*t* = −4.832, *df* = 217.060, *p* < 0.001, *MD* = −3.332), with sufficiently active participants reporting higher life satisfaction.

## Discussion

The COVID-19 pandemic is an unprecedented public health event that presents a significant risk to human health and well-being (19, 20, 30). Efforts to slow the pandemic have required significant changes to “everyday life,” with one unintentional consequence being reductions in PA (22, 46). The purpose of the current manuscript was to investigate whether changes in MVPA during the early stages of the COVID pandemic were associated with mental health outcomes, specifically, depressive symptomology, anxiety symptomology, and life satisfaction.

Overall results suggest a positive association between mental health and change in MVPA among this sample of highly active participants. Individuals who reported larger decreases in MVPA pre- to during-COVID reported relatively poorer mental health as indicated by higher depression and anxiety symptoms, and lower life satisfaction. Between-group analyses found significant differences across mental health categorizations, where individuals with the poorest mental health reported relatively greater changes in MVPA. For instance, with regards to depressive symptomatology, participants who reported no or mild symptoms reported small increases in MVPA. Those with moderate, moderate-severe, and severe symptoms reported reduced MVPA in a gradient fashion that corresponded with symptom severity (Figure 1A). Similar trends were observed with regards to anxiety symptoms and life satisfaction.

Our findings are consistent with those reported in a rapid review that has not yet undergone peer review. Wolf et al. (47) reviewed evidence examining the association between physical activity and depression and anxiety during the COVID-19 pandemic. They identified a total of 21 observational studies (four longitudinal, one cross-sectional with retrospective analysis, and 16 cross-sectional). Their synthesis suggests that people who participated in physical activity on a regular basis with higher volume and frequency and kept their physical activity routines stable in the first few months of the pandemic, showed less symptoms of depression and anxiety. Specifically, those reporting a higher total time spent in moderate to vigorous PA had 12 to 32% lower chances of presenting with depressive symptoms and 15 to 34% lower chances of presenting with anxiety.

A significant body of research conducted prior to COVID indicates a positive relationship between mental health and physical activity (6, 7, 13). The results of the current study and the greater literature (47, 48) suggest that such associations hold true during a period of pandemic and significant socioeconomic disruption. Indeed, given both the extent and severity of disruptions, it is remarkable that a significant proportion of depressive symptomatology – 7.7% of variance accounted for – was associated with MVPA. Though relatively small at an individual level, MVPA may serve as a significant contributor to poor mental health when expanded to the population (49). Additionally, there have been significant and sustained reduction in light physical activity (LPA) during the COVID pandemic (22). LPA is also associated with poor mental health outcomes even when MVPA is controlled for (50), which was not accounted for in the current study. We strongly encourage future research to examine longitudinal associations between all types of physical activity and mental health across the duration of the COVID pandemic.

### The Role of Past Activity

Between-group analyses of sufficiently and insufficiently active individuals revealed an intriguing pattern of results. Whereas, previous research has found MVPA to be protective against psychological distress and mental illness (6, 51–53), participants' retrospectively-reported MVPA did not appear to mitigate mental health symptomatology reported during COVID. That is, participants who were sufficiently vs. insufficiently active *prior to* COVID did not differ in their mental health outcomes *during* COVID. In contrast, significant between-group differences were observed cross-sectionally between sufficiently vs. insufficiently active individuals, with sufficiently active individuals reporting better mental health. The non-significant effect of pre-COVID MVPA may be due to study methodology, in that participants were asked to retrospectively recall their level of physical activity before COVID (54, 55). Alternatively, the COVID pandemic and its resulting impact on mental health (30) may have presented participants with unique biopsychosocial stressors, which dampened the benefits of pre-COVID MVPA on mental health. Further investigation into this observation is warranted.

### Psychological Distress

Within the current study, the majority of participants reported some degree of psychological distress: 62.0% reporting depressive symptoms and 53.7% reporting anxiety. A notable minority reported depressive and/or anxiety symptoms at or above the PHQ-9 and GAD-7 threshold scores of 10: 28.1 and 26.5%, respectively. The occurrence of clinically significant psychological distress in this study is significantly higher than what is typically found in surveys of the general population. For instance, Kocalevent et al. (56) found 5.6% of individuals reported a PHQ-9 score ≥10; whereas Lowe et al. (57) found that 5.1% of individuals reported a GAD-7 score ≥10. Notably, the results from the current study – with a predominantly North American sample – are similar to those reported in China (58) and the United Kingdom (59) during the COVID pandemic. While further investigation is required, the consistency of findings (30) suggest that a five-fold increase in the prevalence of psychological distress may be typical during a period of pandemic.

### Strengths and Limitations

The current paper examines relationships between physical activity and mental health during a period of pandemic in an English-speaking population. Additional strengths include the use of validated clinical mental health measures that included measures of both psychological distress (PHQ-9 and GAD-7) and mental well-being (SWLS), and a validated self-report measure of physical activity. With regards to limitations, participants in this study were primarily younger adults, female, well-educated, from North America, and reported relatively high levels of physical activity (pre- and during-COVID). Consequently, results may not be generalizable to other populations. As this is a cross-sectional study, cause-effect relationships cannot be inferred from results. Additionally, there are a significant number of other factors that may account for poor mental health during the pandemic, such as less socialization, that were not controlled for in the current study. Further research utilizing more diverse samples and a longitudinal design will be useful for understanding associations between physical activity, health, and mental health during COVID.

### Implications for Health and Public Health Professionals

There is growing evidence that the COVID pandemic has negatively impacted both mental health (30, 58, 59) and physical activity (22, 46). The results of this research illustrate a positive association between mental health and MVPA in the early stages of the COVID pandemic, with sufficiently active individuals reporting lower depressive and anxiety symptoms, and higher quality of life. Conversely, individuals with poorer mental health reported greater decreases in MVPA. While the cross-sectional nature of these results prevents examination of causation, the consistency of associations between physical activity and mental health outcomes is noteworthy. Given what is known about the physical (1) and mental health (5–7) benefits of physical activity, it is prudent to promote physical activity involvement as a mechanism for promoting health and well-being during the COVID pandemic. Individuals who maintained their levels of physical activity demonstrated less psychological disturbance. A priority for future research is examining factors that support this resilience. For example, some active adults may have been creative in their home-based leisure activities, using online health and/or physical activity apps, or getting outdoors as much as possible (while following public health requirements). Implementing safe physical distancing measures that provide extra space for everyone to walk or cycle are likely essential. This could include temporary reallocation of roadway space and keeping expansive green spaces open to public access.

## Conclusions

The purpose of the current study was to examine associations between physical activity, mental health, and mental illness during the COVID-19 pandemic. In line with emerging literature (47), individuals who maintained their levels of physical activity demonstrated less psychological disturbance. Results indicate a positive association between changes in MVPA and mental health, where those who experienced the greatest decline in MVPA reported relatively greater psychological distress and lower life satisfaction. Though further research is required to examine longitudinal trends, these early findings speak to the importance of maintaining and promoting physical activity during a period of pandemic. Public health initiatives that support safe physical activity while ensuring physical distancing are likely an important foundation for many adults to initiate or maintain physical activity in the case of future waves of the COVID pandemic, or in the case of future pandemics.

## Data Availability Statement

The data that support the findings of this study are available from the authors upon reasonable request.

## Ethics Statement

The studies involving human participants were reviewed and approved by The University of British Columbia Research Ethics Board (#H20-00899). The participants provided their written informed consent to participate in this study.

## Author Contributions

MG and NR conceived and designed the study and methods. MG and MF cleaned, analyzed, and interpreted the data. MG drafted the manuscript. All authors provided feedback on study design and assisted with participant recruitment and manuscript revisions.

## Conflict of Interest

The authors declare that the research was conducted in the absence of any commercial or financial relationships that could be construed as a potential conflict of interest.

## References

[1] Physical Activity Guidelines Advisory Committee. 2018 Physical Activity Guidelines Advisory Committee Scientific Report to the Secretary of Health and Human Services. Washington, DC: U.S. Department of Health and Human Services (2018).

[2] Hu Y-N Chung Y-J Yu H-K Chen Y-C Tsai C-T Hu G-C. Effect of Tai Chi exercise on fall prevention in older adults: systematic review and meta-analysis of randomized controlled trials. Int J Gerontol. (2016) 10:131–6. 10.1016/j.ijge.2016.06.002

[3] Patel NK Newstead AH Ferrer RL. The effects of yoga on physical functioning and health related quality of life in older adults: a systematic review and meta-analysis. J Altern Complement Med. (2012) 18:902–17. 10.1089/acm.2011.0473 22909385

[4] Yan J-H Gu W-J Sun J Zhang W-X Li B-W Pan L. Efficacy of Tai Chi on pain, stiffness and function in patients with osteoarthritis: a meta-analysis. PLoS ONE. (2013) 8:e61672. 10.1371/journal.pone.0061672 23620778 PMC3631149

[5] Wiese CW Kuykendall L Tay L. Get active? A meta-analysis of leisure-time physical activity and subjective well-being. J Posit Psychol. (2018) 13:57–66. 10.1080/17439760.2017.1374436

[6] Mammen G Faulkner G. Physical activity and the prevention of depression: a systematic review of prospective studies. Am J Prev Med. (2013) 45:649–57. 10.1016/j.amepre.2013.08.001 24139780

[7] Gorczynski P Faulkner G. Exercise therapy for schizophrenia. Cochrane Database Syst Rev. (2010) 2010: CD004412. 10.1002/14651858.CD004412.pub2 PMC4164954 20464730

[8] Stubbs B Koyanagi A Hallgren M Firth J Richards J Schuch F. Physical activity and anxiety: a perspective from the world health survey. J Affect Disord. (2017) 208:545–52. 10.1016/j.jad.2016.10.028 27802893

[9] Rosenbaum S Vancampfort D Steel Z Newby J Ward PB Stubbs B. Physical activity in the treatment of post-traumatic stress disorder: a systematic review and meta-analysis. Psychiatry Res. (2015) 230:130–6. 10.1016/j.psychres.2015.10.017 26500072

[10] Cornelius C Fedewa AL Ahn S. The effect of physical activity on children with ADHD: a quantitative review of the literature. J App School Psychol. (2017) 33:136–70. 10.1080/15377903.2016.1265622

[11] Beckett MW Ardern CI Rotondi MA. A meta-analysis of prospective studies on the role of physical activity and the prevention of Alzheimer's disease in older adults. BMC Geriatr. (2015) 15:9. 10.1186/s12877-015-0007-2 25887627 PMC4333880

[12] Blondell SJ Hammersley-Mather R Veerman JL. Does physical activity prevent cognitive decline and dementia? A systematic review and meta-analysis of longitudinal studies. BMC Public Health. (2014) 14:510. 10.1186/1471-2458-14-510 24885250 PMC4064273

[13] Ravindran AV Lam RW Filteau MJ Lespérance F Kennedy SH Parikh SV. Canadian network for mood and anxiety treatments (CANMAT) clinical guidelines for the management of major depressive disorder in adults.: V. Complementary and alternative medicine treatments. J Affect Disord. (2009) 117:S54–S64. 10.1016/j.jad.2009.06.040 19666194

[14] Canadian Society for Exercise Physiology. Canadian 24-Hour Movement Guidelines. Ottawa, ON: Canadian Society for Exercise Physiology (2020).

[15] U.S. Department of Health and Human Services. Physical Activity Guidelines for Americans, 2nd ed. Washington, DC: U.S. Department of Health and Human Services (2018).

[16] World Health Organization. Physical Activity. Geneva: World Health Organization (2020). Retrieved from: https://www.who.int/news-room/fact-sheets/detail/physical-activity

[17] Katzmarzyk PT Lee I-M Martin CK Blair SN. Epidemiology of physical activity and exercise training in the United States. Prog Cardiovasc Dis. (2017) 60:3–10. 10.1016/j.pcad.2017.01.004 28089610

[18] Colley RC Garriguet D Janssen I Craig CL Clarke J Tremblay MS. Physical activity of Canadian adults: accelerometer results from the 2007 to 2009 canadian health measures survey. Public Health Rep. (2011) 22:7. Available online at: https://www150.statcan.gc.ca/n1/en/pub/82-003-x/2011001/article/11396-eng.pdf?st=IBumGpTg 21510585

[19] Government of Canada. Coronavirus Disease (COVID-19): Outbreak Update. (2020). Available online at: https://www.canada.ca/en/public-health/services/diseases/2019-novel-coronavirus-infection.html#a4 (accessed May, 2020).

[20] World Health Organization. Rolling Updates on Coronavirus Disease (COVID-19). (2020). Available online at: https://www.who.int/emergencies/diseases/novel-coronavirus-2019/events-as-they-happen (accessed May, 2020).

[21] Stockwell S Trott M Tully M Shin J Barnett Y Butler L. Changes in physical activity and sedentary behaviours from before to during the COVID-19 pandemic lockdown: a systematic review. BMJ Open Sport Exercise Med. (2021) 7:e000960. 10.1136/bmjsem-2020-000960 PMC7852071 34192010

[22] Di Sebastiano K Chulak-Bozzer T Vanderloo L Faulkner G. Don't walk so close to me: Physical distancing and physical activity in Canada. Front Health Psychol. (2020) 11:1895. 10.3389/fpsyg.2020.01895 32849110 PMC7396577

[23] Halabchi F Ahmadinejad Z Selk-Ghaffari M. COVID-19 epidemic: exercise or not to exercise; that is the question!: Kowsar. (2020). 10.5812/asjsm.102630

[24] Simpson RJ Katsanis E. The immunological case for staying active during the COVID-19 pandemic. Brain Behav Immun. (2020) 87:6–7. 10.1016/j.bbi.2020.04.041 32311497 PMC7165095

[25] Organization WH. Mental health and psychosocial considerations during the COVID-19 outbreak, 18 March 2020. World Health Organ. (2020).

[26] Jiménez-Pavón D Carbonell-Baeza A Lavie CJ. Physical exercise as therapy to fight against the mental and physical consequences of COVID-19 quarantine: special focus in older people. Prog Cardiovasc Dis. (2020) 63:386–8. 10.1016/j.pcad.2020.03.009 32220590 PMC7118448

[27] Heart and Stroke Foundation. Being Active at Home. Safely. (2020). Available online at: https://www.heartandstroke.ca/articles/being-active-at-home-safely (accessed May, 2020).

[28] Rodríguez MÁ Crespo I Olmedillas H. Exercising in times of COVID-19: what do experts recommend doing within four walls? Rev Esp Cardiol. (2020) 73:527–9. 10.1016/j.rec.2020.04.001 32414660 PMC7142674

[29] CBC News. ‘Please, go Outside': COVID-19 Much Less Likely to Spread Outdoors, Dr. Bonnie Henry Says. Vancouver, BC: CBC British Columbia (2020). Available online at: https://www.cbc.ca/news/canada/british-columbia/please-go-outside-dr-bonnie-henry-says-covid-19-much-less-likely-to-spread-outdoors-1.5550191

[30] Rajkumar RP. COVID-19 and mental health: a review of the existing literature. Asian J Psychiatr. (2020) 52:102066. 10.1016/j.ajp.2020.102066 32302935 PMC7151415

[31] Lesser IA Nienhuis CP. The impact of COVID-19 on physical activity behavior and well-being of canadians. Int J Environ Res Public Health. (2020) 17:3899. 10.3390/ijerph17113899 32486380 PMC7312579

[32] Nienhuis CP Lesser IA. The impact of COVID-19 on women's physical activity behavior and mental well-being. Int J Environ Res Public Health. (2020) 17:9036. 10.3390/ijerph17239036 33291530 PMC7729617

[33] De Moor M Beem A Stubbe J Boomsma D De Geus E. Regular exercise, anxiety, depression and personality: a population-based study. Prev Med. (2006) 42:273–9. 10.1016/j.ypmed.2005.12.002 16439008

[34] Faul F Erdfelder E Lang A-G Buchner A. G^*^ power 3: a flexible statistical power analysis program for the social, behavioral, and biomedical sciences. Behav Res Methods. (2007) 39:175–91. 10.3758/BF03193146 17695343

[35] Sallis JF. Age-related decline in physical activity: a synthesis of human and animal studies. Med Sci Sports Exerc. (2000) 32:1598–600. 10.1097/00005768-200009000-00012 10994911

[36] Azevedo MR Araújo CLP Reichert FF Siqueira FV da Silva MC Hallal PC. Gender differences in leisure-time physical activity. Int J Public Health. (2007) 52:8. 10.1007/s00038-006-5062-1 17966815 PMC2778720

[37] Ladabaum U Mannalithara A Myer PA Singh G. Obesity, abdominal obesity, physical activity, and caloric intake in US adults: 1988 to 2010. Am J Med. (2014) 127:717–27. e12. 10.1016/j.amjmed.2014.02.026 24631411 PMC4524881

[38] Leach LS Christensen H Mackinnon AJ Windsor TD Butterworth P. Gender differences in depression and anxiety across the adult lifespan: the role of psychosocial mediators. Soc Psychiatry Psychiatr Epidemiol. (2008) 43:983–98. 10.1007/s00127-008-0388-z 18575787

[39] Craig CL Marshall AL Sjöström M Bauman AE Booth ML Ainsworth BE. International physical activity questionnaire: 12-country reliability and validity. Med Sci Sports Exerc. (2003) 35:1381–95. 10.1249/01.MSS.0000078924.61453.FB 12900694

[40] Kroenke K Spitzer RL Williams JB. The PHQ-9: validity of a brief depression severity measure. J Gen Intern Med. (2001) 16:606–13. 10.1046/j.1525-1497.2001.016009606.x 11556941 PMC1495268

[41] American Psychiatric Association. Diagnostic and statistical manual of mental disorders (DSM-5®). Arlington, VA: American Psychiatric Pub (2013).

[42] Spitzer RL Kroenke K Williams JB Löwe B. A brief measure for assessing generalized anxiety disorder: the GAD-7. Arch Intern Med. (2006) 166:1092–7. 10.1001/archinte.166.10.1092 16717171

[43] Diener E Emmons RA Larsen RJ Griffin S. The satisfaction with life scale. J Pers Assess. (1985) 49:71–5. 10.1207/s15327752jpa4901_13 16367493

[44] Pavot W Diener E. The satisfaction with life scale and the emerging construct of life satisfaction. J Positive Psychol. (2008) 3:137–52. 10.1080/17439760701756946

[45] Tabachnick B Fidell L. Using Multivariate Statistics. London: Pearson Education (2012).

[46] Fitbit. Fitbit News. (2020). Available online at: https://blog.fitbit.com/covid-19-global-activity/ (accessed May, 2020).

[47] Wolf S Zeibig J Seiffer B Welkerling J Brokmeier L Atrott B. Can physical activity protect against depression and anxiety during the COVID-19 pandemic? A Rapid Systematic Review. (2020) 10.21203/rs.3.rs-81150/v1 PMC8060908 33886101

[48] Caputo EL Reichert FF. Studies of physical activity and COVID-19 during the pandemic: a scoping review. J Physical Activity Health. (2020) 1:1–10. 10.1123/jpah.2020-0406 33152693

[49] Rose G. Sick individuals and sick populations. Int J Epidemiol. (2001) 30:427–32. 10.1093/ije/30.3.427 11416056

[50] Amagasa S Machida M Fukushima N Kikuchi H Takamiya T Odagiri Y. Is objectively measured light-intensity physical activity associated with health outcomes after adjustment for moderate-to-vigorous physical activity in adults? A systematic review. Int J Behav Nutr Physical Activity. (2018) 15:1–13. 10.1186/s12966-018-0695-z 29986718 PMC6038338

[51] Tsatsoulis A Fountoulakis S. The protective role of exercise on stress system dysregulation and comorbidities. Ann N Y Acad Sci. (2006) 1083:196–213. 10.1196/annals.1367.020 17148741

[52] Hegberg NJ Tone EB. Physical activity and stress resilience: considering those at-risk for developing mental health problems. Men Health Physical Activity. (2015) 8:1–7. 10.1016/j.mhpa.2014.10.001

[53] Strawbridge WJ Deleger S Roberts RE Kaplan GA. Physical activity reduces the risk of subsequent depression for older adults. Am J Epidemiol. (2002) 156:328–34. 10.1093/aje/kwf047 12181102

[54] Sallis JF Haskell WL Wood PD Fortmann SP Rogers T Blair SN. Physical activity assessment methodology in the five-city project. Am J Epidemiol. (1985) 121:91–106. 10.1093/oxfordjournals.aje.a113987 3964995

[55] Durante R Ainsworth BE. The recall of physical activity: using a cognitive model of the question-answering process. Med Sci Sports Exerc. (1996) 28:1282–91. 10.1097/00005768-199610000-00012 8897386

[56] Kocalevent R-D Hinz A Brähler E. Standardization of the depression screener patient health questionnaire (PHQ-9) in the general population. Gen Hosp Psychiatry. (2013) 35:551–5. 10.1016/j.genhosppsych.2013.04.006 23664569

[57] Löwe B Decker O Müller S Brähler E Schellberg D Herzog W. Validation and standardization of the generalized anxiety disorder screener (GAD-7) in the general population. Med Care. (2008) 46:266–74. 10.1097/MLR.0b013e318160d093 18388841

[58] Zhang J Lu H Zeng H Zhang S Du Q Jiang T. The differential psychological distress of populations affected by the COVID-19 pandemic. Brain Behav Immun. (2020) 87:49–50. 10.1016/j.bbi.2020.04.031 32304883 PMC7156946

[59] Shevlin M McBride O Murphy J Miller JG Hartman TK Levita L. Anxiety, depression, traumatic stress, and COVID-19 related anxiety in the UK general population during the COVID-19 pandemic. (2020) 6:e125. 10.31234/osf.io/hb6nq 33070797 PMC7573460

